# Sarcoidosis and neuromyelitis optica in a patient with optic neuritis – a case report

**DOI:** 10.1002/acn3.51413

**Published:** 2021-06-24

**Authors:** Michael A. Foster, Sara Collorone, Jacqueline Palace, James Acheson, Ahmed T. Toosy

**Affiliations:** ^1^ Department of Neuroinflammation UCL Queen Square Institute of Neurology London UK; ^2^ Nuffield Department of Clinical Neurosciences John Radcliffe Hospital Oxford UK; ^3^ Strabismus and Neuro‐Ophthalmology Service Moorfields Eye Hospital NHS Foundation Trust London UK

## Abstract

We present a case of atypical recurrent optic neuritis. A man in his 50s presented with right optic neuritis and profound visual loss, associated with elevated inflammatory markers. Lymph‐node biopsy was consistent with sarcoidosis. Aquaporin‐4 antibodies were also present. Three months following corticosteroid treatment, his right optic neuritis relapsed, again with raised inflammatory markers. He was started on azathioprine and prednisolone with good effect. A dual diagnosis of sarcoidosis and neuromyelitis optica with aquaporin‐4 antibodies is very rare. Long‐term immunosuppression is required. The case highlights the importance of identifying the features and cause of atypical optic neuritis.

## Case Presentation

A man in his late 50s presented with a 5‐day history of rapid right eye visual loss associated with pain on eye movement and colour desaturation. Four weeks previously, he had experienced a flu‐like illness with significant weight loss. Past history was significant for factor V Leiden mutation with previous deep vein thrombosis and pulmonary embolism, as well as asthma and chronic obstructive pulmonary disease.

On initial examination, his right eye had no perception of light and his left eye vision was 6/5 on Snellen chart. There was a right relative afferent pupillary defect with a hyperaemic and slightly swollen right disc. Systemic examination was unremarkable.

Erythrocyte sedimentation rate (ESR) was elevated (93 mm/hr). Empirical treatment for giant cell arteritis was started. He was given 500 mg oral methylprednisolone daily for 5 days, followed by 80 mg prednisolone daily. A right temporal biopsy 4 days later was negative.

Anti‐nuclear antibody (ANA) staining was positive at a titre of 1:1280 (homogenous). Peripheral anti‐neutrophil cytoplasmic antibody (pANCA) staining was positive, but myeloperoxidase and proteinase three antibodies were negative. Serum angiotensin‐converting enzyme was normal (32 U/L).

**Figure 1:**
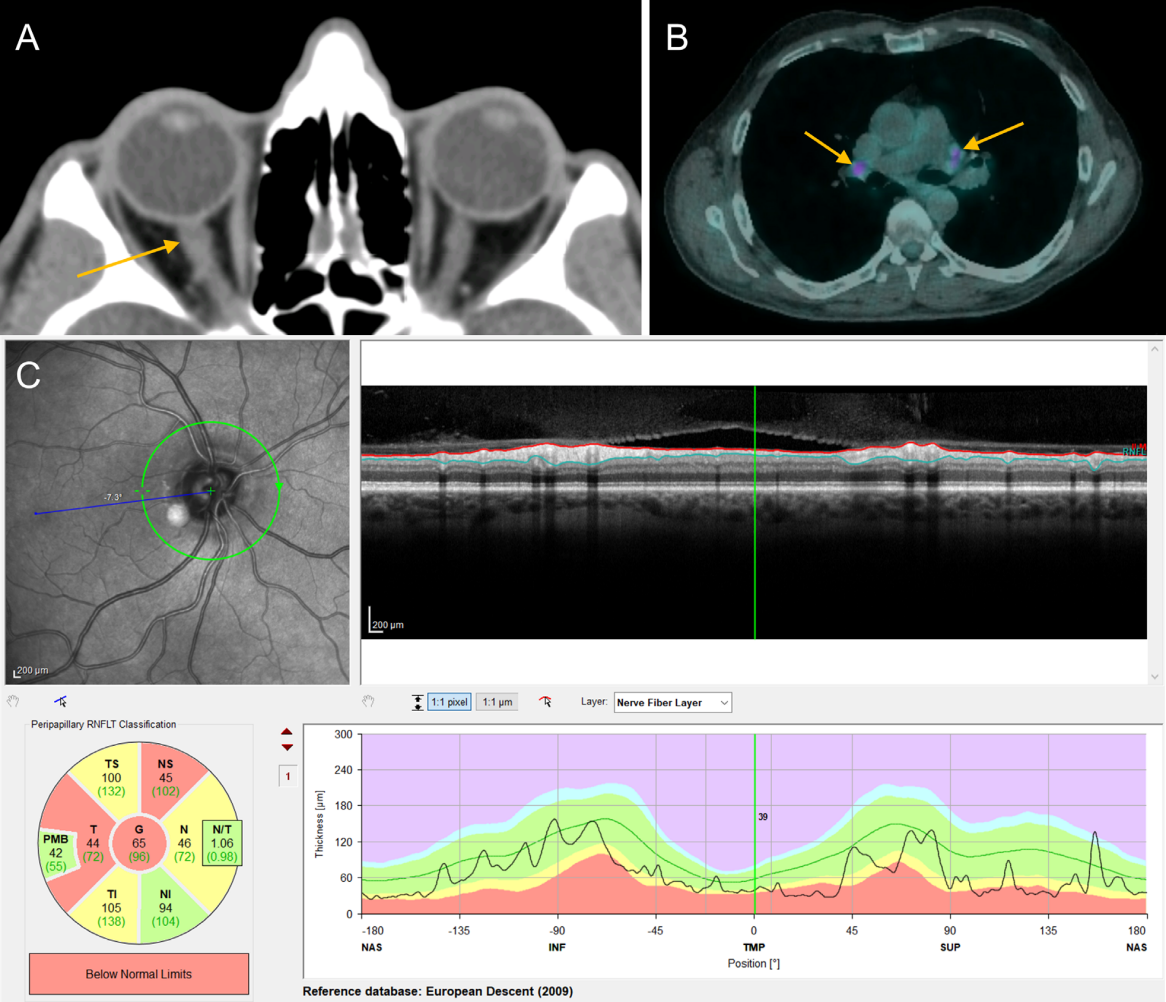
(A) CT head demonstrating asymmetrical enlargement of the optic nerve sheath complex within the right orbit (arrow); (B) FDG‐PET with FDG‐avid hilar lymphadenopathy (arrows); (C) OCT of the right eye, demonstrating supero‐temporal thinning of the peripapillary retinal nerve fibre layer.

CT head identified asymmetrical enlargement of the optic nerve sheath complex within the right orbit (Fig. 1A), with minor streakiness of the surrounding perineural fat. The brain was normal except for the presence of calcification in the basal ganglia, thalami and peridentate cerebellar regions, reported as suggesting a deposition disorder. CT angiography did not show any evidence of vasculitis.

No improvement in vision was seen after 10 days of corticosteroids. A subsequent MRI scan of the orbits was normal and did not show pathological enhancement after contrast, although it was performed after 18 days of corticosteroids. MRI brain confirmed the CT findings in the deep grey matter and cerebellum. He was given 1 g of IV methylprednisolone daily for 3 days, starting on the same day as the MRI.

Visual evoked potentials 3 weeks after presentation revealed severe right optic nerve conduction delay. An 18F‐fluorodeoxyglucose positron‐emission tomography (FDG‐PET) scan of the whole body demonstrated hypermetabolic mediastinal and hilar lymphadenopathy (Fig. 1B). Histology of lymph‐node biopsies demonstrated non‐caeseating granulomata, suggestive of sarcoidosis.

In addition, a live cell‐based assay for aquaporin‐4 (AQP4) antibodies later returned strongly positive. This was confirmed on repeat testing. Live cell‐based assay for myelin oligodendrocyte glycoprotein antibody was negative.

There was a good clinical response to prolonged corticosteroids: at 3 months from onset, ESR had dropped to 18 mm/hr and corrected right‐eye vision had recovered (6/6‐2).

However, 3 months later, whilst weaning corticosteroids, he experienced a relapse of his right optic neuritis (ON) with an elevated ESR (62 mm/hr). Optical coherence tomography showed supero‐temporal thinning of the peripapillary retinal nerve fibre layer in the right eye (Fig. 1C). He was treated with IV methylprednisolone, resulting in symptom improvement, and he was started on azathioprine to reduce relapse risk.

He has been maintained on azathioprine and low‐dose prednisolone with no further clinical deterioration. Right‐eye vision has remained 6/9 with some colour‐vision deficits and infero‐temporal field depression. He has not had other neurological symptoms.

The possibility of a deposition disorder (as suggested by his brain imaging) was thoroughly investigated, but no clear cause was found. This was felt to be an incidental finding not connected to his presentation.

## Discussion

This is a case of atypical ON associated with both histologically proven sarcoidosis and AQP4 antibodies. To date, there is only one case of dual sarcoidosis and neuromyelitis optica spectrum disorder (NMOSD) reported in the literature, presenting with transverse myelitis and later bilateral ON, with both diagnoses made at the second presentation.^1^ In contrast, this case fulfilled diagnostic criteria^2^, ^3^ for both conditions at first presentation (apart from exclusion of other diseases).

It is difficult to conclusively establish whether sarcoidosis or NMOSD was responsible for the recurrent ON. The cell‐based assay for AQP4 antibodies has a specificity of 99%.^4^ However, the greatly elevated ESR is atypical for NMOSD – although lower elevations during relapse are reported^5^ – and might support that sarcoidosis is therefore the more likely cause. The presence of pANCA staining is atypical, though not novel for NMOSD.^6^ However, it should be interpreted with caution when concomitant with strongly positive ANA staining in a homogenous pattern, as in this case.^7^ ANA positivity is often seen in both sarcoidosis and NMOSD.^8^, ^9^

For long‐term management, given the recurrent ON and the presence of AQP4 antibodies, azathioprine was selected due to its efficacy in both conditions.^10^, ^11^ Although treatment of recurrent ON secondary to sarcoidosis can be withdrawn in time if the disease remains stable, the presence of AQP4 antibodies suggests a long‐term course of immunosuppression.

This case highlights the importance of identifying atypical features in ON, such as, in our case, age of onset and marked visual loss.^12^ This prompts a thorough assessment of the diagnosis for the underlying cause so that directed treatment can prevent permanent visual loss. Furthermore, it highlights the importance of interpreting results in the context of the overall clinical picture, even with reliable biomarkers. Finally, it also demonstrates how close follow‐up can refine a differential diagnosis, as further pathological features emerge.

## Conflict of Interests

Michael Foster is supported by a grant from the MRC (MR/S026088/1). Sara Collorone is supported by the Rosetrees Trust (MS632) and she was awarded a MAGNIMS‐ECTRIMS fellowship in 2016. Jacqueline Palace is partly funded by highly specialised services to run a national congenital myasthenia service and a neuromyelitis service. She has received support for scientific meetings and honorariums for advisory work from Merck Serono, Biogen Idec, Novartis, Teva, Chugai Pharma, Bayer Schering, Alexion, Roche, Genzyme, MedImmune, EuroImmun, MedDay, Abide ARGENX, UCB and Viela Bio and grants from Merck Serono, Novartis, Biogen Idec, Teva, Abide, MedImmune, Bayer Schering, Genzyme, Chugai and Alexion. She has received grants from the Multiple Sclerosis Society UK, Guthy Jackson Foundation, National Institute of Health Research, Oxford Health Services Research Committee, Medical Research Council, GMSI (Grant for MS Innovation), John Fell, Myaware and AMPLO for research studies. James Acheson has no competing interests to declare. Ahmed Toosy has received speaker honoraria from Biomedia, Sereno Symposia International Foundation, Bayer and meeting expenses from Biogen Idec and Novartis. He was the UK PI for two clinical trials sponsored by MEDDAY pharmaceutical company (MD1003 in optic neuropathy [MS‐ON] and progressive MS [MS‐SPI2]). He is supported by grants from the MRC (MR/S026088/1), NIHR BRC (541/CAP/OC/818837) and RoseTrees Trust (A1332).

## Author Contributions

Michael Foster drafted and revised the manuscript and performed literature review. Sara Collorone supported in drafting the manuscript and performing literature review. Jacqueline Palace reviewed the manuscript and provided specialist scientific content. James Acheson reviewed the manuscript and provided specialist scientific content. Ahmed Toosy conceived of the report, supervised the project, reviewed the manuscript and provided specialist scientific content.

## Supporting information


**Figure S1**. Fundus photographs taken at first presentation, demonstrating hyperaemia and swelling of the right optic disc.
