# Supplementary figures and images for: Sarcoidosis and neuromyelitis optica in a patient with optic neuritis – a case report
Source: Ann Clin Transl Neurol. 2021 Jun 24;8(8):1760–3. doi: 10.1002/acn3.51413 (PMC8351381; doi:10.1002/acn3.51413)

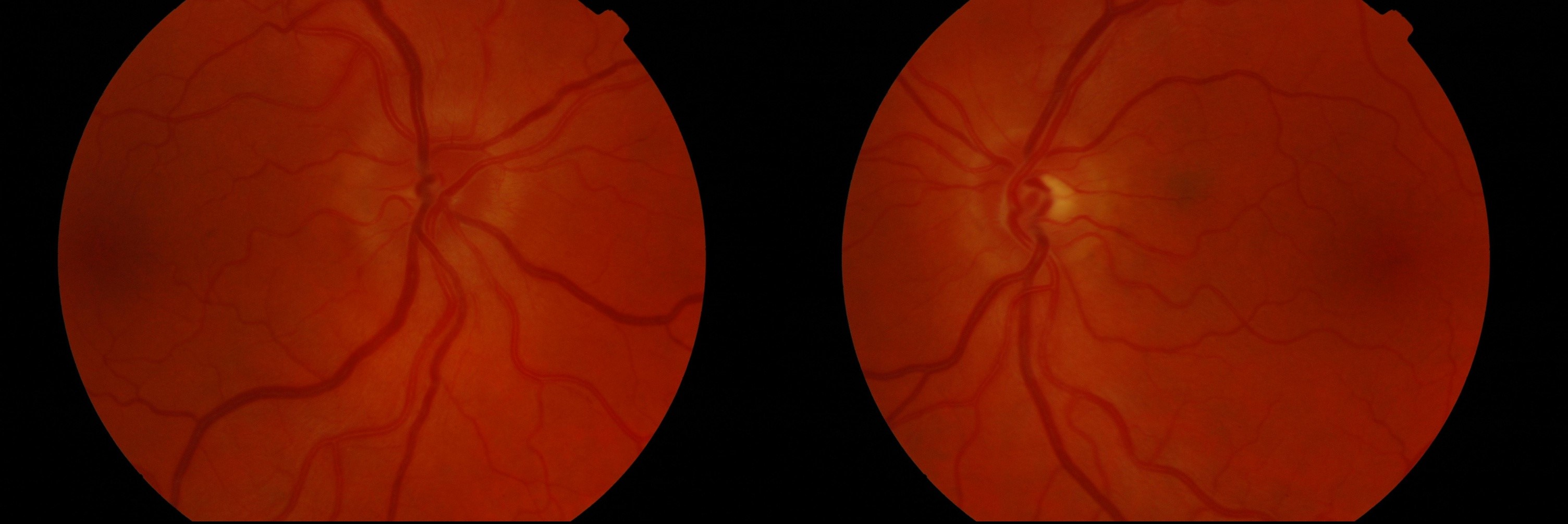

Supplement: Supplementary file 1 — Figure S1. Fundus photographs taken at first presentation, demonstrating hyperaemia and swelling of the right optic disc. [file ACN3-8-1760-s001.png]
